# From insights to innovations: evaluating preclinical paradigms in demyelinating disease therapeutics

**DOI:** 10.1038/s41684-026-01725-6

**Published:** 2026-04-17

**Authors:** Melika Karbalaee, Ally Lin, Luca Peruzzotti-Jametti, Stefano Pluchino, Sabah Mozafari

**Affiliations:** 1 Department of Clinical Neurosciences, National Institute for Health Research Biomedical Research Centre, University of Cambridge, Cambridge, UK; 2 Department of Metabolism, Digestion and Reproduction, Imperial College London, London, UK

**Keywords:** Multiple sclerosis, Multiple sclerosis

## Abstract

Demyelinating disorders such as multiple sclerosis and leukodystrophies are on the rise, posing substantial challenges due to their progressive nature and the current limitations of therapies that effectively restore lost myelin. Over the past decade, advancements in regenerative neuroscience, including cutting-edge stem cell therapies, advanced biomaterials and groundbreaking gene-editing technologies, offer promising avenues for remyelination, immunomodulation and neural repair. Yet, to successfully transition these innovations into clinical therapies, we need robust preclinical models that accurately reflect disease pathology and predict treatment efficacy. This Review offers a thorough overview of the preclinical models utilized in regenerative neurology for demyelinating diseases, highlighting the rapid progress in biomaterial and gene-editing research, which requires meticulous testing and validation in both in vitro and in vivo environments. We begin by explaining the pathophysiology of demyelination, then provide an exhaustive discussion on various preclinical models, including toxin-induced, autoimmune, genetic, viral-induced and large animal models. This is followed by an exploration of emerging regenerative strategies, from cell-based and pharmacological approaches to bioengineered techniques, and we conclude with an analysis of current challenges, translational barriers and future directions in the field. By synthesizing insights from multiple disciplines, this Review strives to engage a diverse audience eager to connect laboratory discoveries with clinical applications in regenerative neuroscience.

## Abstract (web summary)

This Review describes available preclinical models for demyelinating diseases and explores emerging regenerative strategies, while analyzing current challenges, translational barriers and future directions for the field.

## Main

Demyelination disrupts the intricate wiring of the central nervous system (CNS), stripping neurons of their protective myelin sheath and driving progressive axonal loss. Multiple sclerosis (MS)—the most common acquired demyelinating disease—is marked by neuroinflammation, axonal degeneration and accumulating neurological disability^1^. Other acquired disorders, including neuromyelitis optica spectrum disorder and acute disseminated encephalomyelitis, present distinct but equally destructive inflammatory profiles. By contrast, leukodystrophies—genetic disorders of myelin formation and maintenance—such as adrenoleukodystrophy, metachromatic leukodystrophy, Krabbe disease and Pelizaeus–Merzbacher disease (PMD), cause relentless neurodegeneration from early life^2^. These largely human-specific diseases share a unifying hallmark: the loss of myelin integrity, which disrupts neural communication and amplifies maladaptive neuroimmune interactions. Deciphering the mechanisms governing myelin homeostasis, plasticity and intercellular regulation across development, maintenance, adaptation and repair is essential to identify therapeutic targets and halt neurodegeneration.

As myelin provides trophic, structural and metabolic support to axons, prolonged demyelination renders axons highly vulnerable to degeneration. Although remyelination can promote functional recovery and protect axons from degeneration, this process is often inadequate in diseases like MS with frequent episodes of demyelination^3^. Current first-line therapies for demyelinating diseases, including MS, primarily focus on symptom management, mainly through anti-inflammatory and immunomodulatory effects, with their efficacy largely limited to the early stages of the disease^4^. However, these treatments do not address the underlying causes of myelin loss or promote true repair^5^. Consequently, there is an urgent need for regenerative approaches that not only halt disease progression but also promote myelin restoration and functional repair.

Neuroregenerative strategies are advancing to address the root causes of myelin diseases, aiming to restore myelin, suppress inflammation and protect neurons and glia^6^. Preclinical efforts now target the limited repair capacity of the CNS through diverse approaches. Pharmacological agents, such as clemastine fumarate, promote oligodendrocyte precursor cell (OPC) differentiation and enhance endogenous remyelination^7^. Cell-based therapies, using neural stem cells (NSCs), mesenchymal stem cells (MSCs) or induced pluripotent stem (iPS) cell-derived neuroglia and their products, combine immune modulation with tissue repair^8–10^. Tissue-engineering platforms such as hydrogels and scaffolds are being developed to improve cell delivery, reduce glial scarring and create a pro-regenerative niche^11–13^. Neurostimulation techniques, including transcranial magnetic stimulation (TMS) and electrical stimulation, have also shown potential to boost myelin repair and functional recovery in preclinical models^14,15^. However, translating these advances into clinical therapies requires robust preclinical models that closely recapitulate human demyelination for mechanistic studies, efficacy testing and safety validation.

In vitro and ex vivo models, including neuroglia cultures, organoids, organotypic and microfluidic systems, provide controlled platforms to investigate myelination, remyelination and immunomodulation^16,17^. While invaluable for dissecting mechanisms, they cannot fully replicate the complexity of the human brain, particularly immune interactions and long-range connectivity, and they also face limitations in cost, scalability and their ability to model chronic disease progression^18^.

In vivo models capture demyelination and repair in a whole-organism context^19^. These include toxin-induced paradigms (for example, cuprizone (CPZ), lysolecithin)^20^, autoimmune models such as experimental autoimmune encephalomyelitis (EAE) that closely mimic MS pathology^21^, genetic models such as Shiverer mice and PMD^22,23^, viral models^24^ and combined approaches^25,26^. Large animal models, such as nonhuman primates and canines, offer greater anatomical and immunological similarity to humans^9^, but face major drawbacks, including high costs, ethical constraints, long lifespans and disease courses, limited genetic tools and increased variability. Moreover, the adult human CNS has more restricted regenerative capacity than other species, a limitation that worsens with age, underscoring the need for therapies tailored to human-specific biology^27^, particularly in chronic and progressive demyelinating diseases.

This Review critically examines the evolving spectrum of preclinical models in regenerative neuroscience for demyelinating disorders, with a focus on translational relevance. We cover conventional rodent, toxin-, autoimmune-, viral- and genetic-based systems alongside advanced in vitro platforms, including brain organoids, microfluidic devices and iPS cell-derived neuroglia. Special emphasis is placed on humanized and patient-derived models integrating human neural and immune components, enabling precision regenerative approaches. Finally, we explore how innovations in bioengineering, synthetic biology and personalized medicine are reshaping preclinical strategies by building more predictive, clinically meaningful pipelines to accelerate the safe and effective translation of regenerative therapies for demyelinating diseases.

## Pathophysiology of demyelinating disorders

The pursuit of effective treatments has deepened our understanding of human myelin pathology, with ongoing experimental and clinical studies continually refining methods to unravel disease mechanisms and develop targeted therapies. Regardless of the initial trigger, demyelination disrupts axonal saltatory conduction, leading to ionic imbalances, excitotoxicity and impaired nerve signaling, ultimately resulting in axonal damage driven and maintained by neuroimmune activation, loss of glial support, metabolic stress and oxidative injury^28^.

In MS, the most common disabling demyelinating disorder, autoimmune attacks target axoglial elements, causing CNS myelin loss; however, the precise initiating events remain unclear. Two predominant hypotheses have emerged: the ‘outside-in’ model, where peripheral immune activation drives CNS demyelination, and the ‘inside-out’ model, which posits that primary glial or axonal injury triggers the disease. An integrated perspective now links these viewpoints: intrinsic CNS vulnerabilities, such as astroglial metabolic stress, mitochondrial dysfunction and abnormal antigen presentation, may prime the immune system, while systemic immune dysregulation breaches the blood–brain barrier (BBB) and amplifies damage. This bidirectional cycle sustains neuroinflammation, demyelination and axonal degeneration, underpinning the chronic progression of MS^29–31^.

Genetic susceptibility further complicates the picture; genome-wide association studies have identified MS risk variants within the major histocompatibility complex (MHC), notably *HLA-DRB1* alleles, which impair immune tolerance and increase the likelihood of autoreactive lymphocytes infiltrating the CNS^32,33^.

Following inflammatory demyelination, endogenous repair mechanisms such as remyelination and immunomodulation are activated, with NSCs and OPCs having critical roles. However, these processes often falter due to limited regenerative capacity and persistent inflammatory environments. Chronic inflammation particularly affects OPCs, impeding their differentiation and remyelination abilities^29^. Aging exacerbates this challenge: aged OPCs exhibit reduced responsiveness to pro-differentiation cues, and immune cells adopt a more pro-inflammatory phenotype, further impairing repair microenvironments^34–36^.

To study these complex processes, researchers have developed a range of in vitro and in vivo models that replicate different aspects of human demyelinating disease. Simpler models, such as two-dimensional (2D) cell cultures, provide rapid, cost-effective and ethically favorable platforms for screening. Conversely, advanced systems, such as brain organoids, humanized mice and large animal models, more closely mimic human pathology but come with higher costs, complexity and interpretative challenges^37^. Combining multiple models can help overcome individual limitations; nevertheless, increased complexity does not always translate into deeper insights. In some cases, simpler systems are better suited for uncovering fundamental mechanisms^37^. Ultimately, model selection should align with the specific research question and intended application^38^, because no model fully captures the intricacies of human pathophysiology^39^.

Given the central role of preclinical models in understanding myelin pathology and advancing therapeutics, the following sections examine commonly used models in neuroinflammatory demyelinating disease research, highlighting their mechanisms, limitations and recent innovations to enhance translational relevance.

## In vitro and ex vivo models for demyelinating disorders

### Monocultures

Monoculture systems—culturing a single CNS cell type in isolation—remain foundational in neuroinflammation and demyelination research. They enable precise control of the extracellular environment for dissecting cell-intrinsic mechanisms, drug responses and genetic perturbations, although they lack the complexity of multicellular and in vivo interactions. Cells are sourced from rodents, nonhuman primates or humans via embryonic/fetal tissue collection, post-mortem isolation, iPS cells or direct lineage conversion, and maintained in adherent, suspension or bioreactor systems.

#### Primary oligodendroglia

Rodent OPCs and oligodendrocytes—particularly neonatal—are abundant, genetically tractable and reproducible, making them valuable for defining myelin differentiation, inflammatory responses and for near-term pathway discovery and drug screening^40^. Human primary OPCs more closely reflect patient biology, with slower maturation and distinct cytokine sensitivity, but their broader translational use is limited by ethical, availability and scalability constraints^41^. A fundamental limitation of both rodent and human oligodendroglia monocultures is their inability to form compact, multilamellar myelin in the absence of axons, confining their utility largely to differentiation rather than functional myelination studies.

#### Immortalized cell lines

Rodent (OLN-93, CG4) and human (HOG) oligodendroglial lines offer scalability, reproducibility and suitability for high-throughput assays^18^, making them practical for feasibility testing and toxicological assessment; however, their tumorigenic or nonphysiological origins limit maturity and translational relevance.

#### Myeloid cells

Microglia and macrophage monocultures are useful for mechanistic studies of activation states, cytokine profiles and myelin debris clearance^42–44^. However, removal from the CNS microenvironment rapidly alters microglial transcriptional identity, limiting their predictive value for complex in vivo immunomodulatory responses that depend on tissue context and multi-lineage interactions.

#### NSCs and NPCs

Multipotent precursors from rodent subventricular zones or human fetal/iPS cell sources can generate all major CNS lineages, enabling studies on myelination, lineage bias and regenerative therapies^45,46^. Human NSCs retain species-specific maturation kinetics and cytokine sensitivities that improve disease modeling fidelity. However, they remain constrained by variability in differentiation efficiency and long culture timelines.

#### Reprogrammed neural cells

Early studies on generating neural cells primarily used embryonic stem cells. The advent of iPS cell technology has since provided a more accessible and ethically favorable alternative^47^. Somatic sources, such as fibroblasts or peripheral blood cells, can be reprogrammed into iPS cells or directly converted into neural lineages, offering versatile platforms to model MS, PMD and other neuroinflammatory or demyelinating disorders^48–55^. These cells can be differentiated into NSCs, oligodendrocytes, neurons, astrocytes or microglia, enabling the study of patient-specific disease phenotypes while avoiding ethical constraints^56^.

Notably, OPCs derived from iPS cells from patients with MS exhibit upregulated MHC class I transcripts (HLA-A, HLA-B and HLA-C) even in the absence of inflammatory stimulation^57^, highlighting their value for uncovering intrinsic, patient-specific vulnerability states that are difficult to model in animals. These renewable, human-relevant systems enable mechanistic dissection and high-throughput, patient-stratified drug screening. Their strongest near-term utility lies in target discovery and comparative pharmacology rather than as stand-alone predictors of clinical remyelination.

Key limitations include prolonged differentiation timelines^58^, variability in oligodendrocyte yield across iPS cell lines^23^, and partial loss of disease-relevant epigenetic signatures during reprogramming^55^. In addition, iPS cell-derived oligodendroglia often remain developmentally immature and fail to fully acquire adult myelin programs, constraining the modeling of chronic demyelination and long-term regeneration typical of progressive disease. Similarly, iPS cell-derived microglia show reduced transcriptional fidelity to primary human microglia due to the absence of complex CNS cues during differentiation^59^, positioning these systems as exploratory rather than fully predictive immunological models. While iPS cells are foundational for advanced organoid and humanized platforms, these constraints support the use of iPS cell-derived monocultures as complementary components within multimodel pipelines, with maximal translational value achieved through benchmarking against co-culture, ex vivo or in vivo systems that capture axon–glia and immune interactions.

### Co-culture systems

Co-culture models incorporating two or more CNS cell types are widely used in neuroinflammatory myelin disease research to better replicate the intercellular interactions underlying pathology and repair. They enable detailed analysis of neuroimmune crosstalk, intercellular signaling and the influence of one cell type on another’s behavior^60^.

#### Oligodendrocyte lineage cells with neurons

This configuration mimics direct axon–oligodendrocyte or axon–OPC contact, essential for studying myelin sheath formation, axonal metabolic support and bidirectional signaling in myelination^61,62^. It clarifies how neuronal activity influences oligodendrocyte maturation and remyelination^63,64^, and how demyelination impacts neuronal viability^65^. While oligodendrocytes in these systems can ensheathe axons, the lack of a three-dimensional (3D) scaffold often yields loosely wrapped membranes rather than ultrastructurally compact myelin. Consequently, these models are best suited for mechanistic and comparative analyses, and any assertions about myelin compaction should be supported by ultrastructural or biophysical evidence.

#### OPCs with microglia

These cultures mimic the inflammatory milieu of demyelinating lesions^66^. Activated microglia release cytokines and growth factors that can either inhibit or promote OPC proliferation and differentiation^67^, making them key for studying inflammation-driven remyelination dynamics. While useful for probing inflammation–repair coupling, the microglial phenotypic drift observed in culture necessitates cautious interpretation of immunomodulatory findings.

#### OPCs with astrocytes

Astrocytes supply metabolic support, trophic factors and extracellular matrix components influencing myelination^68^. Co-cultures have helped to reveal the roles of astrocytes in both repair and pathology, including scar formation and inflammatory signaling^69–71^. A 2023 study showed that astrocytes derived from isogenic iPS cells carrying the Alzheimer’s-associated ‘C’ allele of the clusterin (*CLU)* rs11136000 single-nucleotide polymorphism exhibit heightened interferon responses and CXCL10 expression compared with astrocytes carrying the nonrisk ‘T’ allele. This inflammatory profile impairs OPC proliferation and myelination^70^, thereby linking astrocytic genotype to white matter vulnerability.

#### Neurons with microglia

Often overlooked in studies focused solely on demyelination, this interaction is, however, critical for understanding the neurodegenerative component of MS that accompanies chronic demyelination. This co-culture model has enabled the study of the microglial effects on neuronal survival, synaptic function and excitability^72^, offering important insight into inflammation-driven neurodegeneration^73^. Such systems are translationally informative for neuroprotection endpoints, but they capture demyelination only indirectly unless integrated with oligodendroglia-containing platforms.

#### Astrocytes with microglia

These co-cultures have enabled the investigation of glial–glial signaling in CNS inflammation^74^, showing how their interplay shapes cytokine profiles, reactive gliosis and the neuroprotective-neurotoxic balance, which in turn influences oligodendrocyte survival, remyelination permissiveness and lesion chronicity in demyelinating disorders.

#### NSCs with myeloid cells or astrocytes

Such systems examine how immune and glial cells influence NSC and neural progenitor cell (NPC) proliferation, differentiation and reparative capacity^75,76^, informing strategies to modulate endogenous repair and glial scar formation^77^. They are therefore well suited for dissecting mechanisms underlying remyelination failure in chronic lesions but require ex vivo or in vivo confirmation, using models in which chronic lesion architecture and vascular/immune dynamics are preserved.

#### Triculture systems

Advances in 2D and semi-3D tricultures—combining neurons, oligodendrocytes, astrocytes, microglia or endothelial cells—enhance the physiological relevance of in vitro myelin and inflammation models^63,78^. These systems can model neurovascular–glial interactions, BBB dysfunction and the combined astrocyte–microglia effects on oligodendrocyte survival and myelination, thereby enabling concurrent assessment of neuronal health, myelin dynamics, immune activation and vascular integrity with high experimental accessibility. However, they still lack the full 3D architecture, extracellular matrix complexity and physiological gradients of living tissue.

Overall, co-culture systems provide greater physiological relevance than monocultures but lack systemic immune and vascular inputs. Variability in media composition and cell ratios can also affect reproducibility and data interpretation, making these systems more suitable for intermediate-stage validation rather than direct translational inference.

### 3D models and bioengineered platforms

Whether based on mono- or co-cultures, 3D platforms bridge the gap between reductionist 2D assays and the structural complexity of living tissue^79,80^. These systems include scaffold-based models (for example, hydrogels^81^ and electrospun fibers^82^), microcarrier-grown cultures and free-floating neurospheres or aggregates maintained in spinner flasks or bioreactors^83^. Such systems enhance nutrient and oxygen delivery, support long-term viability and enable physiologically relevant interactions. By recapitulating key aspects of CNS architecture, including extracellular matrix composition, spatial gradients and organized myelin structures, they provide powerful platforms for mechanistic studies of myelination, demyelination and neuroinflammation, as well as for drug discovery. However, increased 3D complexity often comes at the expense of scalability and standardization, and their translational value depends on the ability to generate reproducible, quantitative endpoints that align with ex vivo and in vivo outcomes.

#### Brain organoids

These models are multicellular 3D structures derived from stem cells that self-organize and differentiate into diverse CNS cell types^17,84,85^. Specialized protocols can be used to produce oligocortical spheroids or myelinoids, enriched in oligodendrocytes^86,87^. Adding promyelinating compounds (for example, ketoconazole, clemastine and T3) increases myelin regulatory factor (MYRF)-positive oligodendrocytes in oligocortical spheroids, making them suitable for remyelination drug screening^88^. However, organoid myelination remains variable and developmentally immature in these systems, showing inconsistent formation of compact myelin and nodal architecture, and typically lacking disease-relevant immune–glial interactions^88,89^. As a result, organoids are best suited for discovery-phase screening and are currently limited in modeling chronic disease progression or supporting late-stage translational validation.

#### Assembloids

These models are created by fusing region-specific brain organoids or combining organoids with specialized glial spheres to model long-range connectivity and multicellular interactions^90^. Examples include cortical–spinal cord assembloids or oligodendrocyte/astrocyte-enriched modules for studying axonal projection, myelination, neuron–glia communication and neuroinflammatory processes in a spatially organized, human-specific context^91^. Despite added anatomical logic, assembloids share key feasibility constraints with organoids, including long culture times and variability, which currently limits broad standardization across laboratories.

#### Microfluidic platforms (organ-on-a-chip)

These platforms are compartmentalized microenvironments that allow precise modeling of neural circuits and cell–cell communication at the microscale^92^. Their applications span nerve injury repair, immune cell trafficking, BBB modeling^93–96^ and demyelination/remyelination assays^97^. A 2025 study integrated iPS cell-derived neuronal and oligodendrocyte spheroids, producing axonal fascicles ensheathed by oligodendrocytes and reproducing key aspects of human myelin biology^98^. However, scalability and adoption remain limited by technical complexity and material constraints, such as polydimethylsiloxane absorption of lipid and hydrophobic compounds essential for myelin formation^99^. Consequently, while well suited for mechanistic microenvironment control and quantitative imaging, their translational utility depends on careful materials selection, assay calibration and interlaboratory reproducibility.

Altogether, despite their promise, 3D in vitro models face technical challenges, such as long culture times, high costs, batch-to-batch variability (notably in organoids) and mostly lack of vascularization, which restricts nutrient/oxygen delivery and causes necrosis in larger constructs. These models represent a major conceptual advance but introduce trade-offs between biological realism, technical complexity and experimental throughput.

To preserve the native architecture, cellular diversity and microenvironment of CNS tissue, ex vivo models provide an intermediate platform between reductionist in vitro systems and complex in vivo models. This makes them particularly valuable for studying mechanisms of demyelination, remyelination and neuroinflammation with spatial and structural fidelity. Compared with in vitro systems, ex vivo models provide higher biological realism (native circuitry, multicellular organization) while retaining experimental control and accessibility; however, feasibility is constrained by tissue availability and culture longevity, particularly for human material.

### Organotypic slice cultures

Rodent CNS slices (200–400 µm) from regions such as the cerebellum^100^, cortex^101^, hippocampus^102^ or spinal cord^103^ preserve intact cytoarchitecture and circuitry. They are widely used to model neurodegeneration, demyelination and neuroinflammation^104,105^, recapitulate axon–glia interactions, perform live imaging of oligodendroglial behavior and test therapeutic interventions to induce (re)myelination^106,107^. By preserving myelinated tracts and local glial networks, slice cultures are well suited for near-term translational triage of promyelinating candidates, particularly when assessing remyelination kinetics and axonal function, yet they lack systemic immune recruitment and peripheral influences.

### Acute slices for electrophysiology and dye-coupling studies

Fresh brain or spinal cord tissues can be used short term to measure conduction changes or calcium signals^108^, synaptic function following demyelination or inflammatory insults or to investigate functional intercellular communications^23^. These preparations are particularly valuable for feasibility-driven functional validation (conduction and network effects), complementing molecular readouts from in vitro systems.

### Explanted preparations

Isolated white matter tracts such as optic nerve or spinal cord maintain native myelinated axons and oligodendrocytes, enabling precise electrophysiological studies of conduction or white matter metabolism^109,110^. Their strength lies in high signal-to-noise functional measurements, but they are limited in modeling chronic immune-mediated demyelination.

### Adult human CNS tissue

Postmortem tissues or surgical resections enable direct study of patient CNS tissue^111,112^, although their availability is limited. Human ex vivo tissue offers the highest face validity for translational inference, but feasibility constraints (sample heterogeneity, postmortem delay and limited throughput) restrict routine use.

Ex vivo models balance physiological complexity with experimental control by preserving native tissue architecture and multicellular interactions while enabling dynamic manipulation, imaging and electrophysiology. However, limited tissue availability, the absence of systemic immune and vascular inputs, and reduced scalability—particularly for human samples—position these models as gatekeepers between in vitro discovery and in vivo validation.

## In vivo animal models

**Fig. 1:**
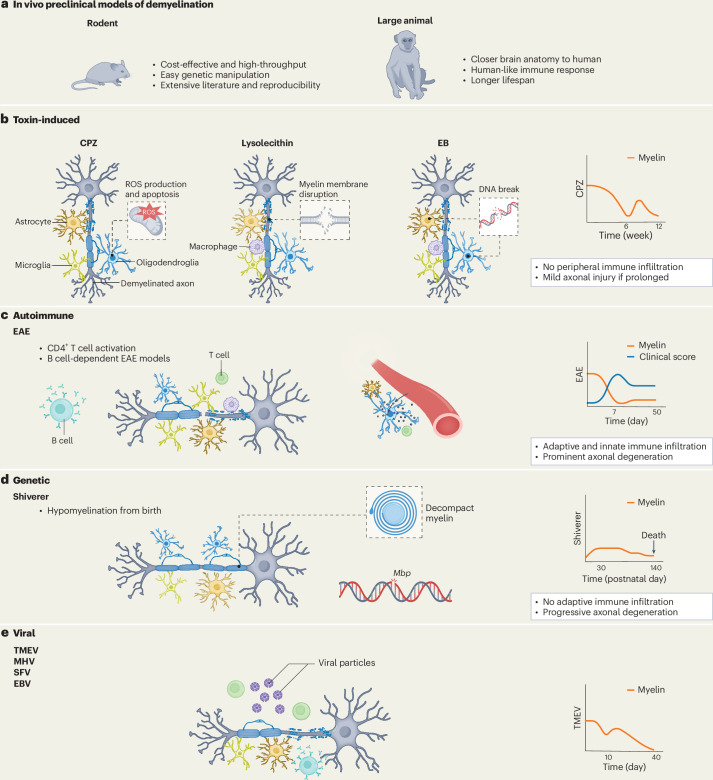
In vivo preclinical models of demyelination. This figure provides an overview of the main in vivo models used to investigate demyelination, categorized into toxin-induced, autoimmune, transgenic and viral approaches. **a**, A comparison of the advantages of rodent versus large animal models is presented, with the latter showing superior translational relevance. **b**, Toxin-induced models include CPZ, which induces demyelination through oligodendrocyte apoptosis and oxidative stress; LPC, which produces focal demyelination by disrupting oligodendrocyte and myelin lipid membranes; and EB, which causes DNA damage and glial cell death. **c**, The autoimmune model is represented by EAE, which models immune-mediated inflammatory demyelination driven by autoreactive T cells and exhibits variable clinical and pathological courses relevant to MS. **d**, The transgenic model illustrated here is the Shiverer mouse, which carries a mutation in the *Mbp* gene encoding myelin basic protein, resulting in severely decompacted and functionally deficient myelin. Other important genetic models include *Plp1*-deficient mice (not shown). **e**, Viral-induced demyelination models include TMEV, MHV, SFV and EBV. These models capture infection-triggered CNS inflammation and demyelination, with TMEV producing chronic progressive demyelination, MHV inducing strain-dependent acute or chronic disease, SFV modeling acute inflammatory demyelination and EBV inducing virus-associated CNS autoimmunity. Schematic timelines at the right of **b**–**e** illustrate the characteristic patterns of myelin loss (and clinical progression for EAE) across key model types.

In vivo models are indispensable for studying demyelination, neuroinflammation and remyelination, as they capture organismal complexity and enable evaluation of therapeutic feasibility, safety and durability. These models include autoimmune, toxin-induced, genetic and viral-induced models (Fig. 1). In translational terms, in vivo systems provide the strongest assessment of feasibility, safety and durability of effect, but no single model reproduces the full clinical spectrum of demyelinating diseases; therefore, model choice should be driven by the specific therapeutic mechanism (immunomodulation versus promyelination versus neuroprotection) and the target disease stage (acute inflammatory versus chronic progressive, early- or late-onset, or age-associated contexts).

### Autoimmune models

EAE is the most widely used model for studying neuroinflammation in MS and other autoimmune CNS disorders^113,114^. It is induced in rodents via active immunization with myelin peptides antigens (for example, MOG_35–55_, MBP_84–104_ and PLP_139–151_) in adjuvants or by adoptive transfer of myelin-specific T cells, producing distinct phenotypes depending on species, strain and protocol^115^. Acute monophasic EAE (Lewis rats, C57BL/6 mice) presents as a single paralysis episode with recovery, ideal for studying early inflammatory demyelination. Chronic progressive EAE (NOD mice) shows sustained decline without remission, modeling neurodegeneration. Relapsing–remitting EAE (SJL/J mice, PLP_139–151_) alternates paralysis and recovery, reflecting MS clinical course^116^. These variants differ in severity, progression and immune mechanisms, guiding model selection^117^. EAE features T-cell-mediated CNS inflammation, demyelination, gliosis and axonal damage, closely resembling MS^118^. Limitations include species-specific immune differences^119^, genetically uniform SPF-raised rodents lacking environmental influences^120^, artificial induction rather than spontaneous onset and mostly diffuse lesions. Targeted EAE, induced by focal stereotactic injection of pro-inflammatory cytokines following systemic immunization with myelin peptides (for example, MOG_1–125_ or MOG_35–55_), produces localized inflammatory demyelinating lesions that enable precise spatial and temporal analysis of lesion evolution; however, the model may lack overt EAE behavioral phenotypes depending on the CNS region targeted^121–123^.

Despite drawbacks, EAE remains essential for studying inflammation-driven myelin loss^124^ and for testing drugs^125^, gene therapies^126^ and cell-based treatments^9,127^. Several approved immunomodulatory therapies, including interferon-β^128^, glatiramer acetate^129^, fingolimod (FTY720)^130^ and anti-α4β1 integrin antibodies (such as natalizumab, which prevents lymphocyte and monocyte accumulation in the CNS^131^), were first validated in EAE. Accordingly, EAE is most predictive for immunomodulatory and immune–CNS trafficking mechanisms, whereas remyelination-specific processes are often obscured by inflammation severity and axonal injury.

### Toxin-induced demyelination models

Various gliotoxins are used to systemically or focally model MS lesions. CPZ, a copper chelator, is most commonly used, inducing oligodendrocyte death, glial activation and demyelination via oral administration. It reproduces key cellular and molecular features of demyelination and remyelination without engaging adaptive immunity. CPZ inhibits mitochondrial complex IV, generating oxidative stress, ROS accumulation and DNA damage in oligodendrocytes^132^, ultimately causing cell death. Depending on treatment duration, the model can be acute (6 weeks) or chronic (12 weeks), with slower spontaneous remyelination after chronic exposure^133^. It has been instrumental for evaluating remyelination-promoting therapies, including the thyromimetic LL-34, which outperformed clemastine^134^, the β-carboline β-CCB, which enhanced GABAergic signaling^135^ and cell-based approaches^136^. Limitations of this model still include the absence of autoimmune components and variability in susceptibility due to animal- and protocol-related factors^137^. Because adaptive immunity is largely absent, the CPZ model is well suited for near-term testing of promyelinating and metabolic-support strategies but is less informative for immune-targeted therapies.

Focal demyelination is commonly achieved using ethidium bromide (EB) or lysolecithin (or lysophosphatidylcholine (LPC)), enabling precise spatial and temporal study of myelin loss and repair^138^. EB, a DNA-alkylating agent, kills astrocytes and oligodendrocytes while sparing axons^139,140^, whereas LPC disrupts oligodendrocyte membranes via detergent-like effects^141,142^. Both agents trigger microglial/macrophage recruitment, astrogliosis, axonal perturbation and OPC mobilization. These models are reproducible and widely used for both mechanistic and therapeutic studies^143^. These models allow precise control of lesion timing and localization for remyelination studies but do not capture the chronic, immune-mediated lesion evolution seen in MS.

### Genetic models

Transgenic myelin mutant models are essential for studying congenital demyelination and evaluating remyelination therapies. The Shiverer mouse, carrying a mutation in the myelin basic protein (*MBP*) gene, lacks MBP, resulting in impaired myelination, severe tremors and neurological deficits from unmyelinated axons^144^. Unlike toxin or EAE models, this model primarily affects oligodendrocytes^145^, allowing focused mechanistic studies. Shiverer mice have been widely used to test OPC and NPC transplantation^146,147^, hydrogel-based cell therapies^11^ and remyelination potential of transplanted gene-edited human OPCs^148^. Genetic models have also advanced research on PMD, an X-linked leukodystrophy caused by *PLP1* mutations^149^. *PLP1* gene duplication creates dosage imbalance, and transgenic mice with extra *Plp1* copies recapitulate demyelination and axonal degeneration, enabling mechanistic studies and therapy testing. Studies in *Plp1*-deficient mice have shown that axonal injury can be driven by cytotoxic CD8⁺ T cells targeting dysfunctional oligodendrocytes carrying *PLP1* defects. Axonal degeneration is reduced when activated microglia efficiently remove the defective myelin^150^. These findings suggest that pathological interactions between axons, dysfunctional oligodendrocytes and immune cells are key drivers of neurodegeneration. Interventions tested in *Plp1* mutant models include NSC transplantation^52^ and gene therapies such as CRISPR–Cas9-mediated knockout of *Plp1* in the jimpy (Plp1^jp^) mouse, a point-mutation mouse model of severe PMD that shows symptomatic improvement following treatment^151^. In addition, antisense oligonucleotides and RNA interference targeting *PLP1* have shown preclinical efficacy and are currently progressing through clinical trials^152^. Thus, genetic models are most directly relevant to leukodystrophies and myelin gene–dose/pathway therapeutics, while extrapolation to inflammatory MS should be made cautiously unless combined with immune-relevant paradigms.

### Viral models

Viral models using Theiler’s murine encephalomyelitis virus (TMEV), mouse hepatitis virus (MHV), Semliki Forest virus (SFV) and Epstein–Barr virus (EBV) are widely used to study MS and postviral demyelinating disorders^24^. TMEV infection in mice triggers an acute encephalitic phase followed by chronic progressive demyelination driven by persistent viral presence and immune activation, modeling aspects of progressive MS, including progressive demyelination. MHV, a coronavirus, induces acute or chronic demyelination depending on strain, allowing investigation of immune-mediated myelin injury and repair. SFV, an alphavirus, models acute inflammatory demyelination and virus-induced glial pathology. EBV, strongly linked to MS risk in humans, has been studied in humanized mice to explore how latent B cell infection may trigger CNS autoimmunity^153^. Limitations of these models include host- and strain-dependent variability, pathogen specificity, biosafety constraints, and difficulties in translating findings to human MS.

### Large animal models

Beyond murine systems, large animals—typically nonhuman primates—provide anatomical, physiological and immunological features closer to humans, enhancing translational relevance^154,155^. Advances in genetic engineering, particularly CRISPR–Cas9, allow precise modeling of human demyelination and neurodegeneration^156^. Nonhuman primates, such as macaques and marmosets, are especially valuable due to their genetic proximity to humans and age- and sex-dependent glial marker patterns that mirror human CNS biology^157^. EAE in marmosets, combined with magnetic resonance imaging and spatial transcriptomics, has revealed the presence of distinct lesion microenvironments and helped to identify a SERPINE1⁺ astrocytic subtype as a secretory hub in perivascular and periventricular regions, which could be potentially critical for lesion initiation^154^. Spontaneous *PLP1*-linked PMD in rhesus macaques reproduces human disease phenotypes, supporting studies on progression and therapy^158^. Canine models, including EB-induced demyelination, allow investigation of progressive MS-like pathology and assessment of remyelination strategies^159^. Large animal models are instrumental for preclinical evaluation of drugs, gene therapies and stem-cell treatments, providing insights into safety, efficacy and chronic disease mechanisms, including progressive MS^9,156^. Despite higher costs and ethical considerations^160^, these models remain indispensable for understanding myelin biology and advancing therapeutic strategies.

### Humanized models

To overcome species-specific differences in immune function and myelination, humanized models have been developed to better recapitulate human/patient CNS biology and evaluate the therapeutic potential of promyelinating candidates^23,161^. Approaches to generate these models include gene editing to express human immune receptors^162^, transplantation of human OPCs^45^ and bone-marrow engraftment to generate mature human immune cells. One model consists of EAE in immunodeficient mice engrafted with EBV-infected human immune cells, which revealed that prior EBV infection intensifies CNS autoimmunity by limiting T regulatory cell expansion and enhancing effector T cell proliferation^163^. In another model, consisting of human–mouse chimeric brains, co-transplanting human iPS cell-derived neural progenitors and primitive macrophage progenitors into neonatal mouse brains enabled the in vivo study of human neuron, astroglia, oligodendroglia and microglia interactions, revealing key pathways such as neurexin–neuroligin and SPP1-mediated signaling^164^. Despite improved human-cell relevance, humanized systems often show incomplete or biased reconstitution (and maturation) of human immune–CNS interactions and typically retain a murine CNS milieu^165^. Consequently, inflammatory demyelination and remyelination dynamics in these models may not fully mirror human lesion evolution.

### Combined models

Toxin-based demyelination and immune-mediated models each have limitations: CPZ lacks inflammation, whereas EAE induces severe axonal damage^166^. In the combined CPZ/EAE (Cup/EAE) model, CPZ pre-treatment enhances immune cell recruitment and lesion formation^26^. The adoptive transfer-CPZ (ATCup) model couples CPZ with myelin-reactive CD4⁺ T cell transfer, inhibiting spontaneous remyelination while preserving axons^167^. Other combined approaches include LPC-induced demyelination in shiverer mice, combining focal toxin injury with congenital myelin deficits^25^. Although combined models capture both injury and immune components, they can introduce nonphysiological ‘double-hit’ effects that alter lesion composition and repair kinetics, complicating the interpretation and translation of the findings.

## Key considerations in model selection

Model selection should be guided by the biological question and translational goal. Different experimental systems provide complementary insights into myelination, demyelination and remyelination. Simpler platforms, such as 2D in vitro cultures, enable rapid, scalable screening with minimal ethical burden, whereas more complex systems, including organoids, provide greater human relevance but introduce challenges in standardization, interpretation and reproducibility^37^. Importantly, increased complexity does not necessarily yield deeper mechanistic insight; reductionist systems often remain optimal for dissecting fundamental pathways^37^. As no model fully recapitulates human disease^39^, cautious interpretation and cross-validation across platforms are essential.

Accordingly, a pragmatic translational pipeline uses 2D monocultures for feasibility screening, co- and tricultures for intercellular validation, 3D systems for human-relevant architecture and quantitative endpoints, and ex vivo and in vivo models for lesion- and organism-level confirmation.

## Preclinical evaluation of regenerative strategies

Advancing regenerative therapies for neuroinflammatory demyelinating diseases requires not only innovative interventions but also appropriate preclinical models that capture the complexity of human pathology and reveal translational potential. Across the different pharmacological, cellular, biomaterial and neuromodulatory strategies, the most impactful studies are those that have aligned model selection with mechanisms central to remyelination, neuroprotection and immune modulation. Importantly, the predictive value of these studies depends on matching each therapeutic mechanism to models that faithfully capture the relevant pathological bottleneck (for example, inflammation, OPC differentiation, axonal vulnerability or repair failure). A summary of key neuroregenerative strategies, their mechanisms and supporting preclinical models is provided (Table 1).

**Table 1:** Summary of key neuroregenerative therapies under investigation for neuroinflammatory demyelination

Therapeutic strategy	Agent/intervention	Mechanism of action	Preclinical model	Model limitation	Suggested tools	Clinical trial
**Pharmacological and small-molecule therapies**	Choline metabolites^174^	↑ OPC proliferation ↑ Remyelination	CPZ and EAE mouse models	Species-specific differences; incomplete progressive MS modeling	Reprogrammed CNS organoids; brain-on-chip	n.d.
	Epidermal growth factor (EGF-GNPs)^169^	↑ OPC differentiation Targeted delivery ↑ Motor coordination and myelin protein expression	CPZ mouse model		Combine with EAE models; brain-on-chip	n.d.
	Anthranilic acid^176^	↓ Pro-inflammatory Th-1 cytokine production	EAE mouse model		Chronic progressive EAE; humanized immune models	n.d.
	Metformin^173^	↓ Inflammation and demyelination	EAE mouse model			Phase I/II/III^206^
	Melatonin^179^	Antioxidant role Anti-inflammatory role	EAE rat model			Phase I/II^207^
	PIPE-307^172^	M1 receptor antagonist ↑ OPC differentiation and myelin repair	EAE mouse model			Phase II (NCT06083753)
	Vitamin D	Antioxidant role ↑ Remyelination	EAE/CPZ mouse models^208^		Humanized immune mouse models	Phase III^209^
			EB mouse models^210^	EB toxin lesions are focal, acute and lack immune contribution		
	Clemastine fumarate^170,171^	↓ M1 receptor ↑ Remyelination Anti-inflammatory role	LPC and EAE mouse models	Species-specific immune differences; variability across models	Humanized immune mouse models; AI-assisted modeling	Phase II (NCT02521311, NCT05131828)
	Betulinic acid^177^	↓ IL-17 and IFN-ɣ cytokine production	In vitro mouse encephalitogenic T cells	Incomplete MS modeling	Humanized immune mouse model; reprogrammed CNS organoids	Phase I/II^178^
	MD1003^161^	↑ Proliferation, ↑ Differentiation of OPCs	Humanized Shiverer mouse model	Shiverer lacks immune-mediated pathology; partial myelin restoration only	Combine with AI-driven modeling	Phase I/II/III^211^
**Cell-based therapies**	NSCs^9,25,48^	Immunomodulation Differentiate into oligodendrocytes and glial cells	Shiverer + LPC mouse; LPC mouse; EAE nonhuman primate model	Species-specific immune and glial differences; variability across models	Experimental demyelination in humanized immune mouse models; AI-driven modeling	Phase I^212,213^
	MSCs^10,136^	Immunomodulation ↑ Remyelination, angiogenesis and axonal repair	EAE and CPZ mouse models			Phase I/II (NCT05532943, NCT05003388, NCT04956744, NCT04749667, NCT05116540)
	OPCs^23,50,51^	Replace myelinating cells ↑ Neurotrophic and immunomodulatory factors	In vitro iPS cell-derived OPC culture; Shiverer (+LPC) mouse models	Lack of chronic neuroinflammation		n.d.
	CAR-T cells^185^	Supress autoimmunity Immunomodulation	EAE mouse model	Species-specific immune differences		Phase I/II (NCT06138132, NCT06451159, NCT06384976, NCT06220201, NCT06617793, NCT06680037)
**Cell-free and biomaterial therapies**	Engineered EVs^186,189^	↑ OPC maturation ↑ Remyelination	In vitro OPC culture; EAE and CPZ mouse model	EV heterogeneity	Standardized EV characterization	Phase I (NCT06620809)
	AAV-mediated gene therapy^126^	↑ Neuronal survival ↑ Remyelination	EAE mouse model	Incomplete MS modeling	Combine with other MS models; reprogrammed CNS organoids	Phase I/II (NCT01801709)
	siRNA^190,192^	↑ Remyelination via gene silencing	EB and LPC mouse models	Variability across models; EB/LPC are focal, acute and lack immune contribution	Combine with EAE models; integrate organoid modeling	n.d.
	Gold nanocrystals^194^	↑ Bioenergetics ↑ Remyelination	Chronic CPZ and LPC mouse models	Lack of immune pathology; variability across models	Humanized EAE mouse model; AI-integrated models	Phase II (NCT03536559, NCT03993171)
**Electromagnetic and neurostimulation therapies**	LI-rTMS^14^	↑ Remyelination ↑ Oligodendrocyte myelination output	CPZ and LPC mouse models	Lack of immune pathology; variability across models	EAE models; AI-integrated models	Phase II^214^
	Vagus nerve stimulation	↓ Neuroinflammation ↑ Myelin preservation	EAE mouse model	Species-specific immune differences	Humanized immune mouse model	Phase I (NCT06641271)

The table outlines the main categories of interventions (small molecules, cell-based therapies, biomaterial/cell-free approaches, neuromodulation and emerging strategies), their primary mechanisms of action and the preclinical models used to test them. Limitations highlight translational barriers such as species-specific immune or metabolic differences, incomplete modeling of progressive MS and variability across models. Suggested solutions reflect recent methodological advances, including humanized immune mouse models, reprogrammed CNS organoids, brain-on-chip technologies, standardized EV characterization and AI-driven modeling, which together aim to improve the predictive validity of preclinical studies for clinical translation. Where applicable, the table also summarizes whether each listed agent/intervention has progressed to clinical trials and the current outcomes. IL-17, interleukin-17; n.d., not determined/not done; PIPE-307; a selective muscarinic M1 receptor antagonist; rTMS, repetitive TMS; Th-1, T helper 1. Upward (↑) and downward (↓) arrows indicate increases and decreases, respectively.

### Small molecules

Pharmacological approaches aim to enhance remyelination by stimulating endogenous stem/precursor cells or modulating neuroimmune interactions^168^. Compounds such as choline metabolites (CDP-choline and citicoline), EGF-coupled gold nanoparticles (EGF-GNP), vitamins, M1 muscarinic receptor antagonists or other neuroprotective molecules have been tested in EAE, CPZ, humanized shiverer or LPC models to promote OPC differentiation and myelin protein expression^161,169–173^. These models differ substantially in their inflammatory load and remyelination kinetics, influencing the interpretability of drug effects.

Citicoline modulates multiple metabolic pathways and oxidative stress in CPZ, EAE and stroke models, improving remyelination and promoting immunomodulation^174,175^. Agents such as anthranilic acid (a synthetic derivative of the tryptophan metabolite anthranilic acid)^176^ suppress CNS inflammation; betulin^177,178^, via the AXL–SOCS3 axis, modulates microglial phenotype and alters disease trajectory^168^. M1 muscarinic receptor antagonists such as clemastine fumarate and PIPE-307 have been developed to enhance OPC differentiation and remyelination^170–172^. Metformin, an antidiabetic agent, has been repurposed as an anti-inflammatory and remyelinating therapy for MS^173^. Neuroprotective strategies such as melatonin supplementation safeguard axons from oxidative stress and mitochondrial dysfunction^179^. A study also evaluated the effects of high-dose biotin (MD1003) on the differentiation potential of human NPCs grafted in the corpus callosum of immunodeficient/myelin-deficient Shiverer mice^161^. Together, these examples illustrate that pharmacological efficacy is strongly context and model dependent and that successful clinical translation requires validation of candidate therapies across complementary paradigms.

### Cell-based therapies

Both EAE and toxin-induced models have been used to dissect the contribution of endogenous or exogenous NSCs, OPCs, NPCs and MSCs to immune regulation, trophic support and direct myelin repair^48,180^. NSCs exert immunomodulatory effects via factor secretion (for example, LIF), mitochondrial transfer and differentiation into oligodendrocytes^181–183^. MSCs shift glial phenotypes from pro-inflammatory to neuroprotective while promoting remyelination and angiogenesis^184^. iPS cell-derived OPCs demonstrate migratory and remyelinating capacity, providing critical insights for the development of autologous therapies^45^. Chimeric antigen receptor T cells (CAR-T) cells show immunomodulation potential by suppressing autoimmunity^185^. Here, inflammatory versus non-inflammatory models are particularly informative for disentangling immune-mediated effects from intrinsic regenerative capacity.

### Biomaterial and cell-free platforms

These strategies extend therapeutic reach while reducing risks such as tumorigenicity. Engineered extracellular vesicles (EVs) and mitochondria promote OPC maturation and remyelination in CPZ, EAE or in vitro models^82,186–188^. Exosomal miR-23a-3p derived from human umbilical cord MSCs was shown to promote remyelination through modulation of the Tbr1/Wnt signaling pathway in vitro using primary rat OPC cultures and in vivo using the EAE mouse model^189^. Adeno-associated virus (AAV)-mediated gene delivery and small interfering RNA (siRNA) targeting (for example, Lingo-1 or NOGO receptors) protect neurons and enhance repair without broad immune suppression^126,190–192^. Biomaterial scaffolds also function as delivery systems and mechanistic probes, enabling targeted interventions in progressive EAE^193^. Gold nanocrystals used in chronic CPZ and LPC mouse models demonstrate neuroprotection and remyelination potential by supporting energy metabolism in oligodendrocytes^194^. These platforms are especially useful for mechanism validation and delivery optimization but require integration with lesion-level models to assess durability and functional recovery.

### Electromagnetic and neurostimulation

CNS activity modulates remyelination. Low-intensity repetitive TMS (LI-rTMS) in CPZ and LPC models promotes myelin internode formation, primarily by enhancing oligodendrocyte integration^14^. Vagus nerve stimulation in the EAE model reduces neuroinflammation and preserves myelin^195^. Careful alignment of stimulation parameters with disease stage and model pathology is essential to distinguish direct glial effects from secondary immune or circuit-level modulation.

**Fig. 2:**
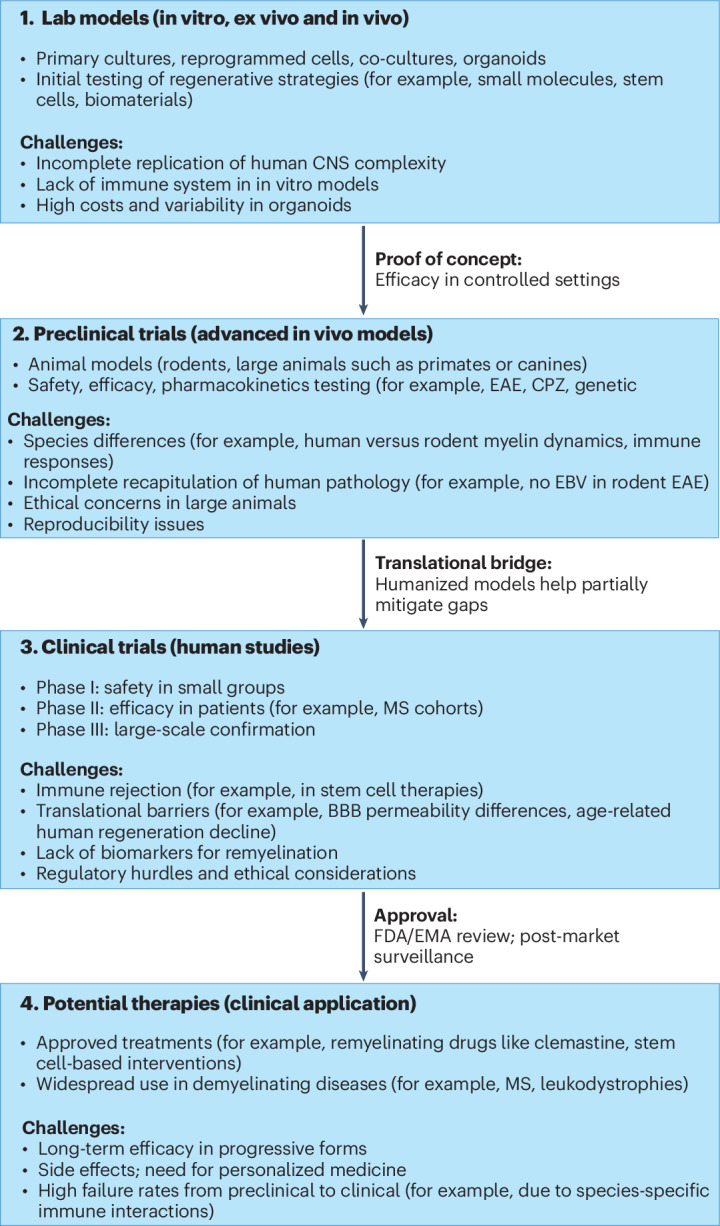
Translational pipeline from preclinical models to clinical trials in neuroinflammatory demyelinating diseases. Schematic overview highlighting the progression from in vitro, ex vivo and in vivo lab models to advanced preclinical testing, human clinical trials and eventual therapeutic applications. Key challenges, translational gaps and regulatory checkpoints are indicated, emphasizing the importance of humanized models and biomarkers to improve clinical relevance and success rates. Clinical trial phases are indicated as follows: phase I (safety in small human cohorts), phase II (efficacy testing in disease-relevant patients, for example, MS cohorts) and phase III (large-scale confirmation of safety and efficacy). EMA, European Medicines Agency; FDA, US Food and Drug Administration.

These studies underscore that therapeutic promise in demyelinating disease is inseparable from strategic model selection. CPZ, LPC and EAE models each expose distinct regenerative bottlenecks; pairing interventions with model pathobiology strengthens mechanistic insight and derisks clinical translation (Fig. 2).

## Bridging preclinical gaps for regenerative therapies

Despite substantial advances in animal modeling, the preclinical pipeline for neuroinflammatory demyelinating diseases such as MS faces critical translational barriers. Below, we outline key gaps, including specific model limitations that directly impact therapeutic interpretation and translational decision-making (summarized in Box 1).

Box 1 Challenges in translating preclinical findings to patients Preclinical models are essential for advancing neurogenerative therapies in demyelinating diseases like MS, but considerable barriers hinder translation to human patients. These challenges contribute to high failure rates in clinical trials and underscore the need for more human-relevant models. We provide here a summary of key barriers limiting the predictive value of current preclinical models, including species differences, immune disparities, incomplete disease recapitulation, ethical and practical concerns in large animal use and lack of standardization and regulatory alignment:

**Species differences and translational barriers**

Fundamental differences between rodents/large animals and humans (for example, myelin structure, BBB permeability and immune system components such as CD8^+^ T cells in human MS versus CD4^+^ in mouse EAE models) limit predictive accuracy. Rodent models often fail to replicate human-specific factors such as EBV involvement or age-related declines in regenerative potential, leading to poor extrapolation of drug efficacy and safety.

**Immune rejection and response disparities**

Therapies such as stem cell transplants or gene editing may trigger immune rejection in humans, which is not fully captured in animal models lacking human immune components (for example, absence of a functional human immune system in in vitro organoids or rodent EAE). This can result in unforeseen inflammatory responses or reduced efficacy in progressive MS forms.

**Incomplete recapitulation of human disease**

No single model fully mimics the complexity of human pathology (for example, EAE focuses on inflammation but neglects remyelination; toxin models like CPZ lack autoimmune elements and clinical symptoms). This leads to gaps in understanding chronic progression, remyelination failure and neuroimmune interactions.

**Ethical and practical challenges in large animal studies**

Models using nonhuman primates or canines offer better human relevance but raise ethical concerns (for example, animal welfare), high cost (challenging genetic manipulations) and logistical issues (for example, longer lifespans for chronic studies). These factors limit scalability and accessibility.

**Lack of standardization, reproducibility and regulatory gaps**

Variability in model protocols (for example, CPZ dosing depends on eating behavior) and insufficient biomarkers for outcomes such as remyelination complicate data interpretation. Regulatory hurdles, such as proving safety before human trials, often delay progress, emphasizing the need for humanized models and artificial intelligence (AI)-driven refinements.

Addressing these through interdisciplinary efforts such as patient-derived organoids and multimodal therapies could potentially enhance translation and accelerate clinical applications.

Toxin-based models such as CPZ primarily induce demyelination without immune components, while autoimmune models such as EAE capture inflammatory aspects but insufficiently address remyelination or chronic progression. ‘Organoids’ refer to reprogrammed patient-derived 3D CNS cultures, used to model remyelination and neuroimmune interactions in vitro.

### Species-specific barriers to CNS drug delivery

Core BBB architecture, including tight junction integrity, is largely conserved between rodents and humans; however, interspecies differences in solute carrier and efflux transporter expression and kinetics can substantially affect CNS drug exposure and brain penetration estimates. This distinction is particularly relevant for the delivery of small molecules (such as iron chelators^196^) and biologics (for example, stem cells^197^) whose efficacy depends on sustained CNS exposure.

Human iPS cell-derived BBB-on-chip and 3D bioprinting platforms now recreate the neurovascular unit, including endothelial cells, pericytes and astrocytes, within microfluidic systems that mimic shear stress and transport dynamics^94–96,197,198^. These models provide high-throughput, human-relevant platforms for the screening of CNS-active compounds, enabling early elimination of poorly penetrant candidates and optimization of in vivo dosing strategies to reduce clinical trial failures.

### Immune repertoire mismatch

Human MS lesions are dominated by immune components such as cytotoxic CD8⁺ T cells, EBV-infected B cells and *CD28*^null^ T cells, which are absent in the conventional EAE model. This dissimilarity limits the predictive value of standard EAE for therapies targeting human-specific immune mechanisms. Humanized immune mouse models address this gap by reconstituting immunodeficient mice (for example, NSG) with human immune components via (1) peripheral blood mononuclear cell transfer for rapid T and B cell engraftment, (2) hematopoietic stem cell transplantation for broad, self-renewing immunity, or (3) grafting human lymphoid tissues to promote immune maturation. These models may yield functional human immune compartments, enabling in vivo modeling of MS-relevant immunopathology. A 2024 study showed that peripheral blood mononuclear cell-humanized B2m-NOG mice (HLA-DRB1*15 MS donors) develop spontaneous and EAE-induced hCD8⁺ T cell-driven CNS lesions, closely resembling MS pathology; however, this model remains limited by poor monocyte engraftment, absence of demyelination, disorganized lymphoid structures and deficient IgG responses^199^. Complementarily, organoid-based neuroinflammation-on-chip platforms allow precise, human cell-based control of immune–CNS interactions, facilitating detailed investigation of processes such as microglial–endothelial crosstalk.

### Capturing progressive MS and remyelination failure

EAE primarily models acute inflammatory episodes, whereas classical toxin-based models (CPZ and lysolecithin) enable detailed cellular investigation of demyelination and repair but fail to reproduce chronic neurodegeneration and complex lesion evolution characteristic of progressive MS^38^. To bridge this gap, 3D human oligocortical organoids derived from patient-specific NSCs, OPCs, astrocytes or microglia have been proposed to study demyelination, axonal injury and remyelination failure in a genetically and clinically relevant context^198^. Interestingly, aged rodents in the CPZ model show delayed and incomplete remyelination, with sustained demyelination after toxin withdrawal, thereby mimicking progressive MS-like repair failure^200^. Finally, fully humanized multilineage CNS mouse models integrate human adaptive immunity, microglia and oligodendrocytes, allowing induction of EAE-like or toxin-mediated demyelination within a functional humanized immune system. These systems may uniquely enable the study of patient-specific immune–CNS interactions, remyelination failure mechanisms and candidate neuroprotective or myelin-promoting therapies under inflammatory conditions that better mirror the progressive form of neuroinflammatory demyelination.

### Reproducibility

Variability in toxin models, dosing regimens, lesion localization, quantification methods and behavioral readouts limits cross-study comparability^201,202^. Adoption of standardized, open-access protocols and centralized reference datasets is essential to harmonize preclinical workflows. The use of AI-driven image analysis, which leverages computer vision to automatically detect and quantify myelin damage, can help to further reduce observer bias and enhance interlaboratory consistency^203^. Addressing standardization, reproducibility, and regulatory gaps—including defining harmonized endpoints and validated biomarkers—will improve the clinical relevance of preclinical demyelination research.

### Ethical and practical constraints

Large animal models better approximate human neuroanatomy and white matter architecture, but ethical and logistical limitations restrict their use^204^. These systems are best reserved for late-stage validation of safety, delivery and durability rather than early discovery. Alternatives include swine iPS cell-derived CNS organoids and human–BBB combined systems, which offer physiological relevance and scalability, as well as ex vivo nonhuman primate brain slice cultures that provide anatomically accurate environments without the logistical or ethical challenges of in vivo studies.

### Emerging strategies

Synthetic biology and nanotechnology are converging to enhance model utility and therapeutic development. Engineered OPCs resistant to inhibitory lesion cues improve remyelination in LPC models, while EV-based nanocarriers—loaded with transferrin, mitochondria, microRNAs or small molecules—enable precise delivery of promyelinating cargo in focal, CPZ or systemic EAE paradigms^148,187,188^. AI-driven computational modeling and in silico screening integrate imaging, histological and genomic datasets to guide experimental design, prioritize drug candidates and predict therapeutic outcomes^205^. These tools support personalized therapeutic development and accelerate translation by integrating patient-specific variables and simulating complex demyelination–repair dynamics.

Collectively, these innovations signal a shift from reliance on singular, imperfect models toward multiplatform, human-integrated strategies. By combining advanced in vitro, in vivo and computational systems, the field can better capture the immunological, cellular and bioenergetic complexity of MS. This new approach enables more rational model selection, reduces translational attrition and strengthens the predictive power of preclinical studies to advance regenerative therapies toward the clinic.

## Conclusion

This Review integrates current insights into the immune, neuroglial and metabolic mechanisms driving myelin damage and impaired repair in neuroinflammatory demyelinating diseases, with a focus on MS.

We examined a wide range of preclinical models—from in vitro systems and classical toxin- and immune-mediated paradigms to humanized mice, large animal models and patient-derived 3D CNS organoids—highlighting their complementary roles in mechanistic discovery and therapeutic testing. Together, these models are essential for evaluating immunomodulatory, neuroprotective and remyelination strategies, particularly for progressive MS where treatment options remain limited.

Key translational challenges persist, including incomplete modeling of human lesion immunopathology, limited representation of chronic neurodegeneration, interexperimental variability and ethical or logistical constraints. Emerging solutions such as AI-assisted imaging, standardized protocols, engineered glial cells, EV-based delivery systems and computational disease modeling, are improving model fidelity, reproducibility and translational relevance.

Looking ahead, integrative approaches combining humanized immune models with engrafted human glia, engineered BBB systems and CNS organoids offer promising avenues to capture patient-relevant inflammatory, degenerative and repair processes. By integrating in vitro, ex vivo, in vivo and in silico platforms, preclinical pipelines can better reflect MS complexity, enhance predictive power and accelerate the development of effective regenerative therapies.
